# Formulation, Characterization, and In Vitro Drug Release Study of β-Cyclodextrin-Based Smart Hydrogels

**DOI:** 10.3390/gels8040207

**Published:** 2022-03-26

**Authors:** Muhammad Suhail, Quoc Lam Vu, Pao-Chu Wu

**Affiliations:** 1 School of Pharmacy, Kaohsiung Medical University, Kaohsiung 80708, Taiwan; u108830004@kmu.edu.tw; 2 Department of Clinical Pharmacy, Thai Nguyen University of Medicine and Pharmacy, 284 Luong Ngoc Quyen Str., Thai Nguyen 24000, Vietnam; vuquoclam@tump.edu.vn; 3 Department of Medical Research, Kaohsiung Medical University Hospital, Kaohsiung 80708, Taiwan; 4 Drug Development and Value Creation Research Center, Kaohsiung Medical University, Kaohsiung 80708, Taiwan

**Keywords:** hydrogel, β-cyclodextrin, sol–gel analysis, dissolution studies

## Abstract

In this study, novel pH-responsive polymeric β-cyclodextrin-*graft*-poly(acrylic acid/itaconic acid) hydrogels were fabricated by the free radical polymerization technique. Various concentrations of β-cyclodextrin, acrylic acid, and itaconic acid were crosslinked by ethylene glycol dimethacrylate in the presence of ammonium persulfate. The crosslinked hydrogels were used for the controlled delivery of theophylline. Loading of theophylline was conducted by the absorption and diffusion method. The fabricated network of hydrogel was evaluated by Fourier transform infrared spectroscopy (FTIR), thermogravimetric analysis (TGA), X-ray diffractometry (XRD), and scanning electron microscopy (SEM). The crosslinking among hydrogel contents and drug loading by the fabricated hydrogel were confirmed by FTIR analysis, while TGA indicated a high thermal stability of the prepared hydrogel as compared to pure β-cyclodextrin and itaconic acid. The high thermal stability of the developed hydrogel indicated an increase in the thermal stability of β-cyclodextrin and itaconic acid after crosslinking. Similarly, a decrease in crystallinity of β-cyclodextrin and itaconic acid was observed after crosslinking, as evaluated by XRD analysis. SEM revealed an irregular and hard surface of the prepared hydrogel, which may be correlated with strong crosslinking among hydrogel contents. Crosslinked insoluble and uncrosslinked soluble fractions of hydrogel were evaluated by sol–gel analysis. An increase in gel fraction was seen with the increase in compositions of hydrogel contents, while a decrease in sol fraction was observed. Dynamic swelling and dissolution studies were performed in three various buffer solutions of pH 1.2, 4.6, and 7.4, respectively. Maximum swelling and drug release were observed at higher pH values as compared to the lower pH value due to the deprotonation and protonation of functional groups of the hydrogel contents; thus, the pH-sensitive nature of the fabricated hydrogel was demonstrated. Likewise, water penetration capability and polymer volume were evaluated by porosity and polymer volume studies. Increased incorporation of β-cyclodextrin, acrylic acid, and itaconic acid led to an increase in swelling, drug release, drug loading, and porosity of the fabricated hydrogel, whereas a decrease was detected with the increasing concentration of ethylene glycol dimethacrylate. Conclusively, the prepared hydrogel could be employed as a suitable and promising carrier for the controlled release of theophylline.

## 1. Introduction

Theophylline (THP) is a bronchodilator drug used in the management of chronic obstructive pulmonary disease [1,2]. The biological source of THP is the leaves of Camellia sinensis. The intake of THP leads to relaxation of bronchioles and muscles of pulmonary sanguine vessels that determines the bronchodilator and relaxing behavior of THP upon the smooth muscles [3]. The reported half-life of THP is 8 h, which decreased to 5 h in smoking patients. Hence, in order to avoid fluctuations and maintain a constant plasma concentration, THP dose needs to be taken multiple times in a day [4], which leads to certain complications, including nausea, vomiting, abdominal pain, insomnia, jitteriness, and irregular or fast heartbeat. Furthermore, patient compliance is also decreased [5]. Hence, to overcome all these complications, different drug carrier systems were developed in order to prolong the release of THP for an extended period of time. Zhang et al. (2008) prepared chitosan/β-cyclodextrin-based microspheres by the spray drying method for sustained release of theophylline [6]. Likewise, Ahirrao and his coworkers fabricated sodium alginate based hydrogel beads for sustained delivery of theophylline [7]. Still, the challenges faced by rapid administration of THP are not overcome. Therefore, due to the unique properties of hydrogels such as high hydrophilicity, stability, biocompatibility, biodegradability, and low toxicity, they are considered the most suitable carrier agent for controlled delivery of therapeutic agents. Hence, the authors have prepared β-cyclodextrin-based hydrogels for controlled delivery of THP to avoid the adverse effects generated as a result of THP intake. 

Hydrogels are crosslinked three-dimensional polymeric hydrophilic networks, which absorb a high quantity of water without losing their structural configuration. The use of hydrogels has been increased, especially in pharmaceutical and biomedical fields, due to their unique properties such as high stability, exceptional swelling index, drug loading, biocompatibility, degradability, biodegradability, and gelling capabilities [8,9]. Due to good response to external stimuli such as temperature, pH, magnetic fields, electric fields, and ionic strength [10,11], a special class of hydrogels which is known as stimuli-sensitive hydrogels has gained a great interest in drug delivery systems. pH-sensitive hydrogel, which is the most studied and important type of stimuli-sensitive hydrogels, plays an important role in controlled and targeted drug delivery systems [12]. 

β-Cyclodextrin (β-CD) is an acyclic oligosaccharide, having both hydrophobic and hydrophilic cavities. Both β-CD and γ-CD are considered safe chemicals by US Food and Drug Administration (FDA) and are employed for various purposes. A key role is played by β-CD on both industrial and pharmaceutical levels as it is easily available and economical. It has various cavity dimensions that are suitable for a number of drug candidates [13]. Acrylic acid (Aa) is a synthetic monomer that has the potential to swell highly in water due to the presence of –COOH groups. These functional groups of Aa bring changes in the ionic strength as the pH of the medium is changed. At low pH values, protonation of Aa occurs, whereas at high pH values, deprotonation of Aa occurs. Hence, protonation and deprotonation of functional groups of Aa influence the swelling of hydrogels as an increase in swelling is observed with the deprotonation of –COOH groups while almost a low swelling is perceived with the protonation of –COOH groups [14]. Itaconic acid (Ia), a synthetic monomer, plays an important role in controlled drug delivery systems [15]. The main capability of Ia is its ease of copolymerization, which generates –COOH side chains of the polymer chain. These functional groups have hydrophilic character and possess the capability to interact with other equivalent groups through hydrogen bonding. The hydrophilic nature of Ia is due to its two –COOH groups with altered pKa values. Therefore, a small quantity of IA is enough to show response to the external stimulus (pH) and enhance the swelling index of hydrogels [16,17]. Moreover, the stability, stiffness, and mechanical strength of hydrogels could be enhanced by the incorporation of comonomers, which could contribute further to H– bonding [18].

The current study is based on the preparation of pH-responsive hydrogels of β-cyclodextrin for controlled delivery of theophylline. The novelty of the presented study is the incorporation of two hydrophilic monomers, i.e., acrylic acid and itaconic acid, with β-cyclodextrin polymer. The sensitivity of the developed hydrogel was enhanced by both acrylic acid and itaconic acid, which enabled the developed hydrogel to swell highly at high pH values as compared to low pH values. The advantage of the current fabricated hydrogel is the protection of the drug from the acidity of stomach and also the protection of the stomach itself from the adverse effects of the drug. Hence, due to maximum swelling at pH 7.4, a high amount of drug was released at basic pH of 7.4 in a controlled way by the fabricated hydrogel. The prepared hydrogels were processed for further investigations. 

## 2. Results and Discussion

### 2.1. Synthesis of β-CD-g-P(Aa/Ia) Hydrogels

**Figure 1:**
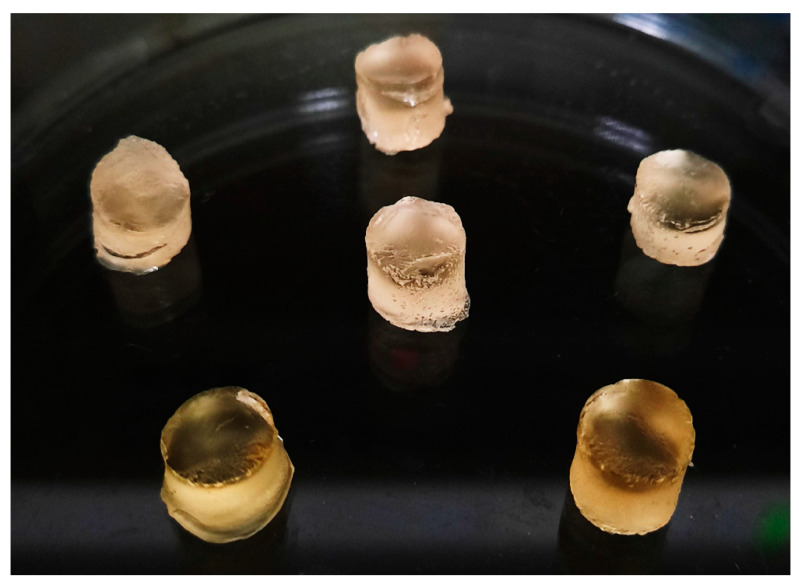
Physical appearance of prepared β-CD-*g*-P(Aa/Ia) hydrogel.

Various concentrations of polymer (β-CD), monomers (Aa, Ia), and crosslinker (EGDMA) were employed for the development of β-cyclodextrin-*graft*-poly(acrylic acid/itaconic acid) (β-CD-*g*-P(Aa/Ia)) hydrogels by the free radical polymerization technique, as shown in Table 1. The polymerization of all formulations was carried out in a water bath at a temperature of 55 °C for an initial 2 h. The temperature was enhanced later up to 65 °C for the next 22 h. The prepared hydrogel discs were then placed in a vacuum oven at 40 °C for drying. After dehydration, the discs were subjected to a series of studies. The physical appearance of dried hydrogel is given in Figure 1.

### 2.2. Fourier Transform Infrared Spectroscopy (FTIR)

**Figure 2:**
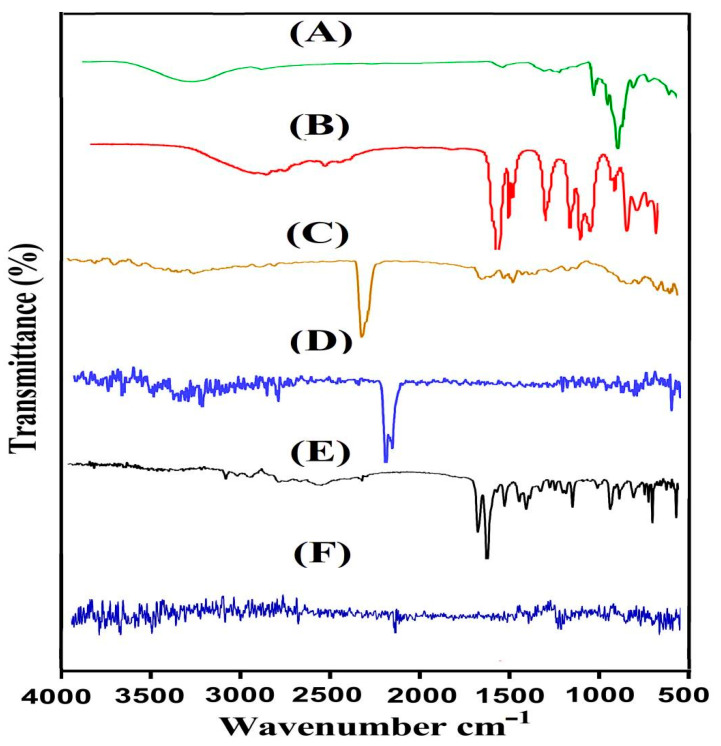
FTIR spectra of (**A**) β-CD, (**B**) Aa, (**C**) Ia, (**D**) unloaded β-CD-*g*-P(Aa/Ia) hydrogel, (**E**) THP, and (**F**) loaded β-CD-*g*-P(Aa/Ia) hydrogel.

The preparation of β-CD-*g*-P(Aa/Ia) hydrogel was confirmed by the evaluation of FTIR spectroscopy of β-CD, Aa, Ia, and fabricated hydrogels, as indicated in Figure 2. FTIR spectrum of β-CD (Figure 2A) presented stretching vibration of methylene C−H and ether C=O by peaks at 2972 and 1022 cm^−1^, respectively, whereas a peak at 3308 cm^−1^ indicated stretching vibration of alcoholic O−H [13]. Similarly, Aa revealed FTIR spectra (Figure 2B) with peaks at 2887, 1673, and 1604 cm^−1^, which were assigned to stretching vibration of –CH_2,_ C–O, and C–C. Likewise, the peak at 1201 cm^−1^ indicated stretching vibration of –C–O group [19,20]. The characteristic peaks of Ia were revealed by FTIR spectra (Figure 2C) at 1656, 1627, and 1480, which were assigned to stretching vibration of C=O, C=C, and C–O–H, respectively. Two peaks at 1228 and 1007 cm^−1^ indicated stretching vibration of the C–O group and out-of-plane banding of the OH group, respectively [21,22]. Certain peaks of polymer and monomers were changed due to the crosslinking and chemical interaction among hydrogel contents, as indicated in Figure 2D. The prominent peaks of β-CD were changed from 1022 and 2972 cm^−1^ to 1040 and 2950 cm^−1^ peaks of the developed hydrogel. Similarly, a few peaks of Aa and Ia were moved from 1201 and 2887 cm^−1^ and 1007 and 1228 cm^−1^ to the 1270 and 2950 cm^−1^ and 1030 and 1310 cm^−1^ peaks of the formulated polymeric network of the hydrogel. A few peaks of β-CD, Aa, and IA disappeared, while certain new peaks were formed. Hence, the shifting, disappearance, and formation of new peaks revealed the successful grafting of Aa and IA over the backbone of β-CD, which led to the development of a new polymeric network of the hydrogel. The FTIR spectral analysis of THP is indicated in Figure 2E and revealed stretching vibration of C=O, C=C, and C–O by peaks at 1566, 1537, and 1178 cm^−1^, respectively. A fluctuation in certain peaks of THP was observed in drug-loaded hydrogels (Figure 2F). The peaks at 1178 and 1537 cm^−1^ were moved to 1205 and 1490 cm^−1^ peaks of the drug-loaded hydrogel. This change in peaks of THP was because of drug loading by the developed hydrogel, and hence no chemical interaction of drug with hydrogel contents was observed [23]. 

### 2.3. Thermogravimetric Analysis (TGA)

**Figure 3:**
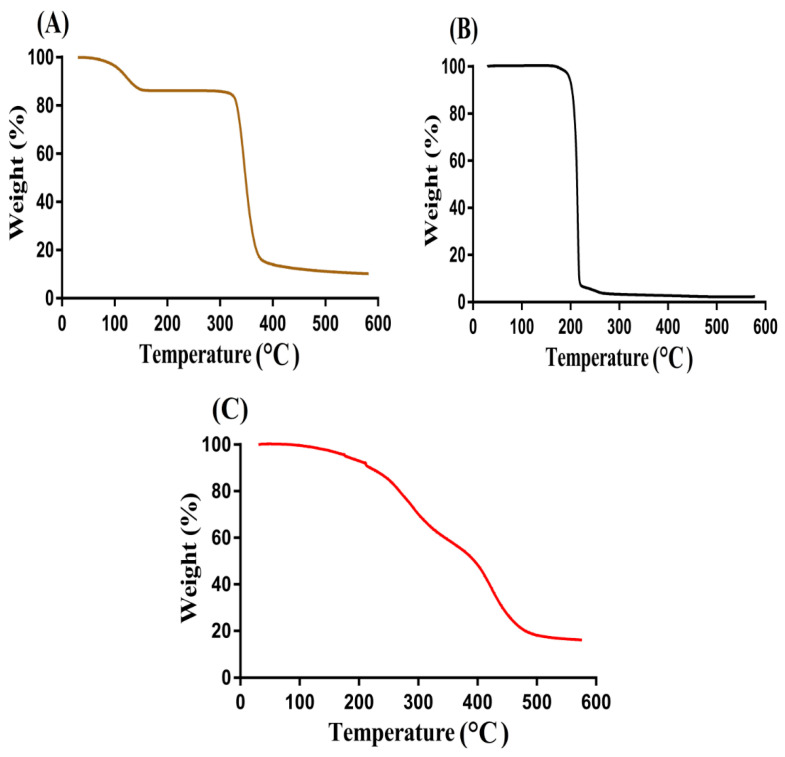
TGA of (**A**) β-CD, (**B**) Ia, and (**C**) β-CD-*g*-P(Aa/Ia) hydrogel.

TGA was performed in order to determine the thermal stability of pure polymer, monomer, and developed hydrogel, as shown in Figure 3. A weight reduction of 12% was detected initially at 116 °C due to loss of bound water by TGA thermogram of β-CD (Figure 3A). Further decrease in weight was observed with the increase in temperature, and almost 63% weight reduction was perceived as the temperature approached 380 °C. Increased temperature led to degradation of β-CD [24]. Like β-CD, weight reduction of Ia occurred with the increase in temperature (Figure 3B). A 90% reduction in weight of Ia was detected as the temperature reached 223 °C due to the elimination of water and anhydride ring formation. A slight 4% weight reduction was observed at 278 °C. After that, due to the carbonization and decarboxylation process, degradation of IA started, which continued until complete paralysis [25]. Similarly, the TGA thermogram of fabricated hydrogel (Figure 3C) demonstrated a slight reduction of 8% in weight at 205 °C related to water loss of β-CD. After that, a reduction of 72% in weight was seen with the increase in temperature up to 488 °C. Further increase in temperature led to degradation of formulated hydrogel, which continued until entire paralysis. Hence, comparing the thermal stability of pure polymer and monomer with developed hydrogel, we can conclude that the thermal stability of both polymer and monomer was less than that of the developed hydrogel. An increase in thermal stability of β-CD and Ia occurred due to crosslinking and polymerization process among hydrogel contents, as indicated by the TGA thermogram of the fabricated hydrogel. The high thermal stability of the developed polymeric network of hydrogel presented strong intermolecular interaction of hydrogel contents, which occurred as a result of grafting, crosslinking, and polymerization reaction [26,27,28]. Nasir and her coworkers prepared Pluronic F127 based gels for controlled delivery of ivabradine hydrochloride and reported an increase in thermal stability of polymer after crosslinking with other gels’ contents, which further supports our hypothesis [29].

### 2.4. X-ray Diffraction Studies (XRD)

**Figure 4:**
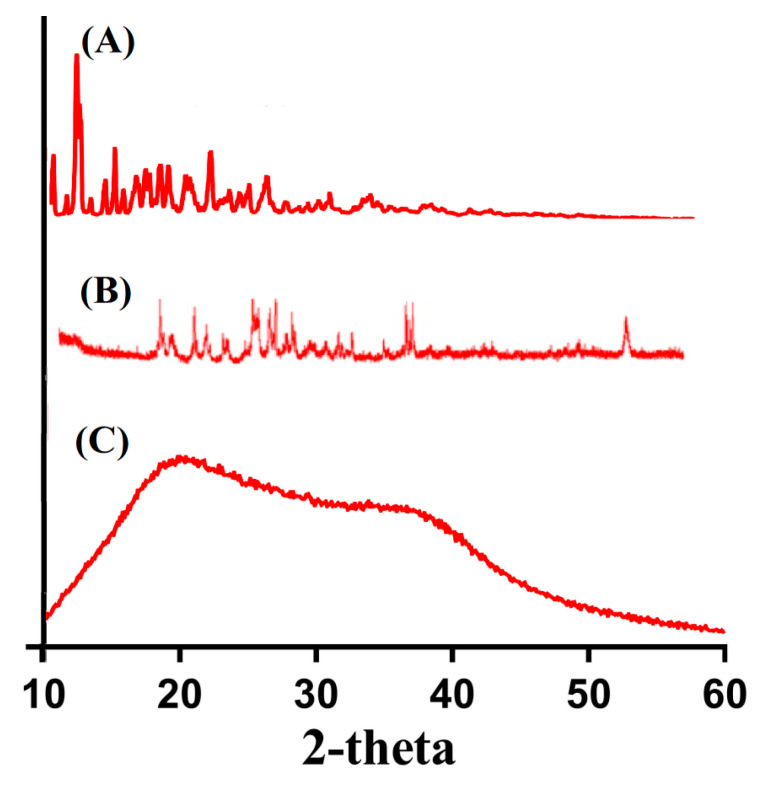
XRD of (**A**) β-CD, (**B**) Ia, and (**C**) β-CD-*g*-P(Aa/Ia) hydrogel.

The physical nature of β-CD, Ia, and developed hydrogel was evaluated by XRD analysis, as shown in Figure 4. The sharp and highly intense peaks of a compound indicate its crystalline nature, while diffuse peaks presented amorphous nature. The XRD pattern of β-CD (Figure 4A) exhibited sharp, highly intense, and prominent crystalline peaks at 2θ = 11.80°, 15.75°, 21.50°, and 27.90°. Similarly, XRD analysis of Ia (Figure 4B) indicated sharp and crystalline peaks at 2θ = 18.90°, 21.60°, 25.78°, 37.82°, and 54.45°. The sharp, crystalline, and highly intense peaks of β-CD and Ia were reduced or disappeared due to crosslinking and grafting, as indicated in the XRD analysis of the developed network of hydrogel (Figure 4C). The highly intense, crystalline, and sharp peaks of β-CD and Ia were replaced by low-intensity peaks, which demonstrated the successful crosslinking, grafting, and polymerization of hydrogel contents due to which crystallinity of pure reagent was reduced [30]. Abdullah et al. (2018) developed polyvinyl alcohol based hydrogel and demonstrated a decrease in crystallinity of polyvinyl alcohol by fabricated hydrogel [31].

### 2.5. Scanning Electron Microscopy (SEM)

**Figure 5:**
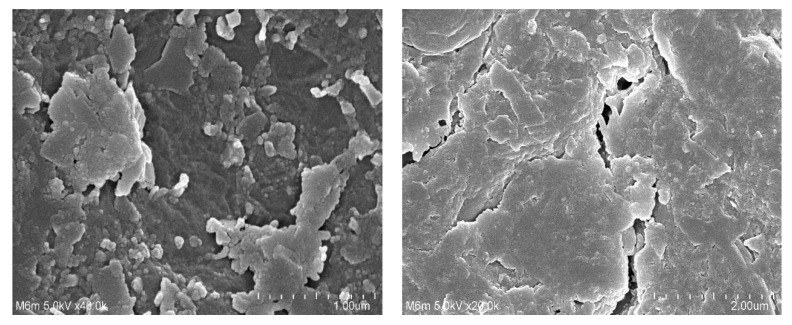
Scanning electron microscopy of β-CD-*g*-P(Aa/Ia) hydrogel.

The surface morphology of fabricated hydrogel was investigated by SEM, as shown in Figure 5. An irregular and hard surface was seen in the SEM examination of the formulated hydrogel. Large cracks and wrinkles could be seen, which may be correlated to the partial collapsing of gel during the process of dehydration. The hard surface of polymeric hydrogel revealed the strong intermolecular interaction that existed among the various contents of the fabricated hydrogel after the polymerization process [32]. 

### 2.6. Sol–Gel Fractions

**Figure 6:**
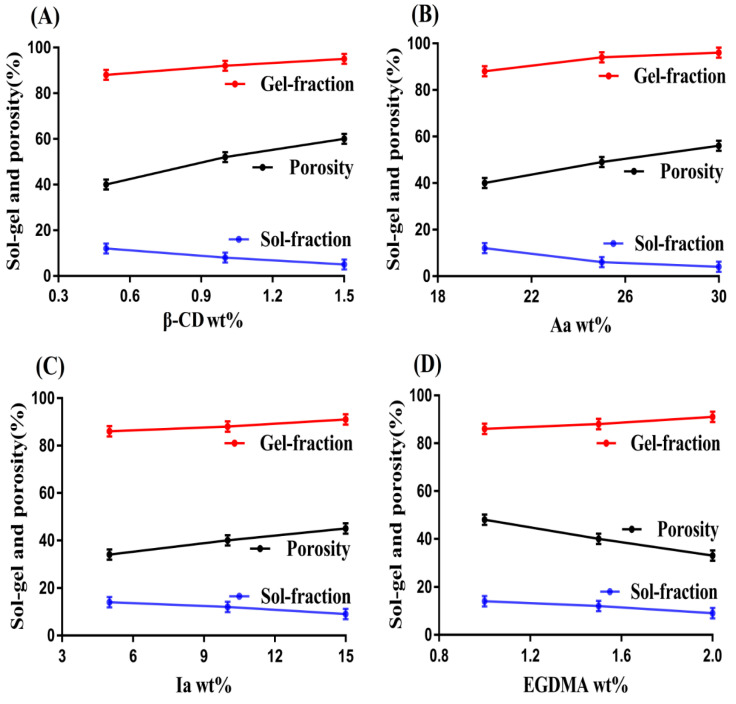
Effect of (**A**) β-CD, (**B**) Aa, (**C**) Ia, and (**D**) EGDMA on sol–gel and porosity of β-CD-*g*-P(Aa/Ia) hydrogel.

Sol is the soluble uncrosslinked fraction, while gel is the insoluble crosslinked fraction of hydrogel. Due to the inverse relationship between the gel and sol fractions, an increase in one content leads to a decrease in other content. Both sol and gel fractions were influenced by the various feed ratios of hydrogel contents, i.e., β-CD, Aa, Ia, and EGDMA, as indicated in Figure 6A–D. An increase in the feed ratio of β-CD led to an increase in the gel fraction. The reason was the availability of a greater number of free radicals, which were generated highly with the increasing feed ratio of β-CD. Hence, a greater concentration of β-CD will increase the generation of free radicals for polymerization and crosslinking of hydrogel contents, thus increasing the gel fraction. Like β-CD, the gel fraction was increased with the increased incorporation of Aa and Ia contents. A high number of free radicals were generated with the increasing concentration of both Aa and Ia for the polymerization of β-CD content, which led to rapid polymerization of hydrogel contents; thus, as a result, an increase in gel fraction was detected. Similarly, bulk and crosslinking densities of the fabricated hydrogel were increased with the increase in feed ratio of EGDMA. The pore size of hydrogel was decreased due to high crosslinking, and thus an increase in gel fraction was observed. Khanum and her coworkers prepared polymeric HPMC-g-poly(AMPS) hydrogel and reported an increase in gel fraction with the increase in feed ratio of polymer, monomer, and crosslinker, which further supports our hypothesis [33]. Unlike the gel fraction, a decrease in the sol fraction was observed [34] as the feed ratio of β-CD, Aa, and Ia was increased and vice versa. 

### 2.7. Porosity Study

A porosity study was conducted for all formulations of β-CD-*g*-P(Aa/Ia) hydrogel. The main purpose of this study was to evaluate the penetration capability of a fluid through the pores into the hydrogel network. Porosity was influenced by the various combinations of hydrogel contents as an increase in porosity was seen with the increasing composition of β-CD, Aa, and Ia, as indicated in Figure 6A–D. The reason may be the highly viscous nature of the reaction mixture, which was formed due to the polymerization of hydrogel contents. The increasing concentration of β-CD, Aa, and Ia content caused the formation of a highly viscous mixture, and as a result, evaporation of bubbles was restricted. This led to the generation of interconnected channels, and thus the porosity of the hydrogel was increased. Contrary to what was observed for the polymer and monomers, a decrease in porosity was observed as the feed ratio of EGDMA was increased. The decrease in porosity due to the increasing composition of EGDMA was due to the formation of a highly crosslinked bulk network of hydrogel, due to which the pore size of developed hydrogel was decreased. Thus, a limited number of channels were generated, and hence, a decrease in penetration and porosity was observed. Thus, we can conclude from the discussion that the higher the porosity, the greater the swelling and drug loading [35].

### 2.8. Swelling Study

**Figure 7:**
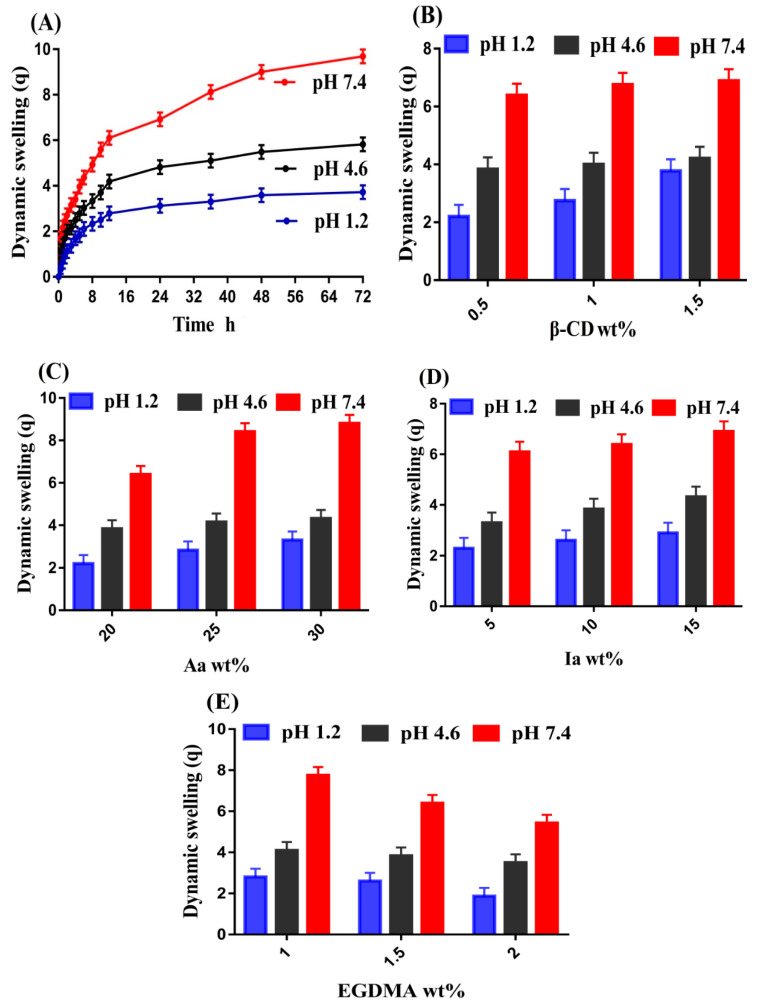
Effect of (**A**) pH, (**B**) β-CD, (**C**) Aa, (**D**) Ia, and (**E**) EGDMA on dynamic swelling of β-CD-*g*-P(Aa/Ia) hydrogel.

A swelling study was performed for the β-CD-*g*-P(Aa/Ia) hydrogel in order to investigate the response of prepared hydrogel at pH 1.2, 4.6, and 7.4, as indicated in Figure 7. Almost low swelling was observed at pH 1.2 as compared to pH 4.6 and 7.4, which indicated the pH-sensitive nature of the fabricated hydrogel (Figure 7A). The low and high swelling of prepared hydrogel was due to the protonation and deprotonation of –COOH functional groups of both monomers, i.e., Aa and Ia. During protonation of –COOH groups, conjugates were formed with counterions through strong hydrogen bonding, due to which the charge density of –COOH groups was decreased, and as a result, low swelling was exhibited at pH 1.2. A change was seen in the swelling of the developed hydrogel as the pH changed from 1.2 to 4.6 and 7.4. Due to the deprotonation of –COOH groups at pH 4.6 and 7.4, the charge density of the same groups was increased, and as a result, strong electrostatic repulsive forces were generated. These forces counteracted each other, and thus maximum swelling at pH 7.4 was observed as compared to pH 4.6. Hence, we can conclude that swelling of fabricated hydrogel was perceived in an order of pH 7.4 > 4.6 > 1.2 [36,37,38].

The swelling of formulated hydrogel was affected by various combinations of β-CD, Aa, Ia, and EGDMA. The swelling was increased with the increased incorporation of polymer and monomer contents (Figure 7B). The –OH and CH_2_OH functional groups of β-CD were produced highly with the increase in its composition, due to which charge density increased. Thus, an increase in swelling was observed [39]. Similarly, an increase in the swelling index of fabricated hydrogel was seen with the increasing composition of Aa and Ia (Figure 7C,D) due to the generation of a high number of –COOH groups. These functional groups of both monomers exerted strong electrostatic repulsive forces, which led to expansion in the volume of hydrogel, and thus an increase in swelling was perceived [40,41,42]. Unlike what was found for the polymer and monomers, a reduction in the swelling of hydrogel was found with the increasing composition of EGDMA (Figure 7E). The crosslinking and bulk densities of the formulated hydrogel were increased, due to which the pore size of the hydrogel was decreased; thus, as a result, water penetration into the polymeric hydrogel was decreased. This hard network and less penetration of water led to a decrease in swelling with the increasing composition of EGDMA [43,44,45,46]. 

### 2.9. Polymer Volume Fraction

The determination of polymer volume fraction for developed hydrogel was carried out at three various pH values, i.e., pH 1.2, 4.6, and 7.4, as shown in Table 2. Polymer volume was found greater at pH 1.2 as compared to pH 4.6 and 7.4. Hydrogel contents, i.e., β-CD, Aa, Ia, and EGDMA, highly affected the polymer volume fraction at all pH values. A decrease was observed in polymer volume fraction with the increase in the composition of polymer and monomers. i.e., β-CD, Aa, and Ia. The main reason is attributed to the high swelling of hydrogel, which was achieved due to an increase in the concentration of polymer and monomers. Unlike what was observed for the polymer and monomers, an increase in polymer volume fraction was observed with the increasing composition of EGDMA, which is attributed to the low swelling of hydrogel. The low and high polymer volume values at pH 7.4, 4.6, and 1.2 indicated a high swelling index of the fabricated hydrogel at high pH values [47].

### 2.10. Drug Loading 

The amount of drug loaded by the formulated hydrogel was evaluated by the extraction and weight methods as indicated in Table 2. Drug loading depends on the porosity and swelling index of the hydrogel. A greater porosity allows a higher penetration capability of a medium into the hydrogel network and thus greater swelling and drug loading. Hence, we can conclude there is a direct relation between the swelling and drug loading [48]. Like porosity and swelling, the loading of a drug by the fabricated hydrogel was also influenced by the various compositions of β-CD, Aa, Ia, and EGDMA. An increase in drug loading was detected with the increasing composition of β-CD, Aa, and Ia. A high number of functional groups of polymer and monomers were generated with their high composition, which led to greater charge density and repulsive forces, and thus an increase in swelling and drug loading was observed. Contrary to what was observed for the polymer and monomers, drug loading was decreased as the concentration of EGDMA was enhanced. The reason is the low porosity and swelling, due to which a decrease in drug loading was perceived [49]. 

### 2.11. Drug Release Studies

**Figure 8:**
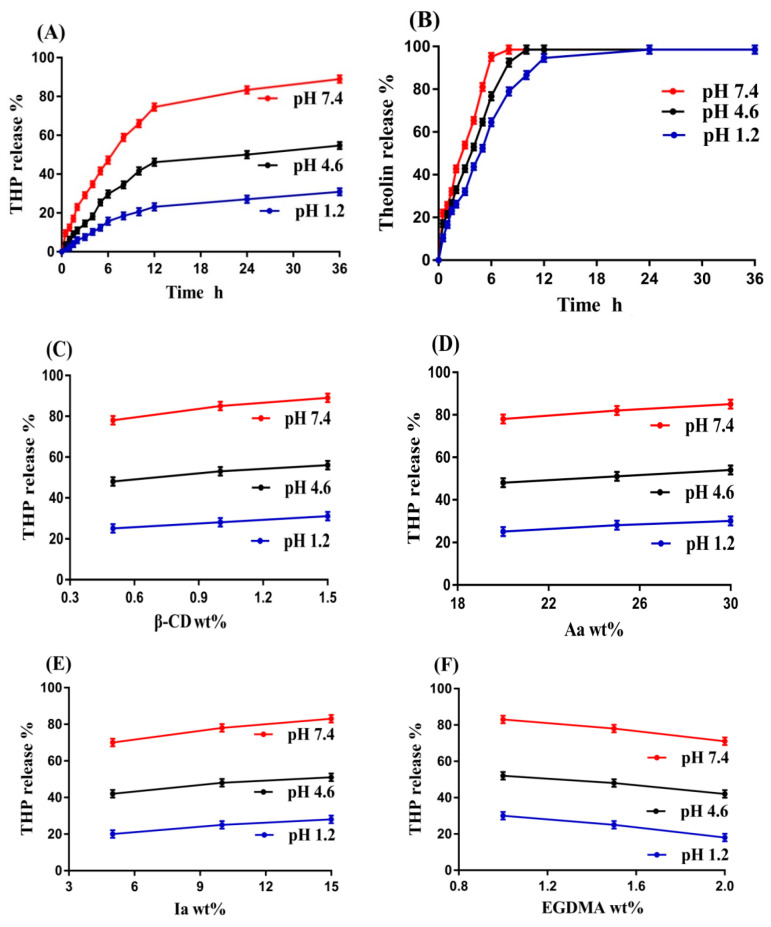
Effect of pH on (**A**) percent drug release from β-CD-*g*-P(Aa/Ia) hydrogel; (**B**) percent drug release from Theolin tablets; and effect of (**C**) β-CD, (**D**) Aa, (**E**) Ia, and (**F**) EGDMA on percent drug release from β-CD-*g*-P(Aa/Ia) hydrogel.

In vitro drug release studies were performed for β-CD-*g*-P(Aa/Ia) hydrogel in order to evaluate the pH-responsive nature of the fabricated hydrogel at three pH values, i.e., pH 1.2, 4.6, and 7.4. Maximum drug release was achieved at pH 7.4 as compared to 4.6, while a very low drug release was perceived at pH 1.2, as shown in Figure 8A. Like swelling, protonation and deprotonation of COOH groups of both monomers occurred, which led to low and high release of drug from the fabricated hydrogel. Due to deprotonation, the charge density of COOH groups was increased very highly at pH 7.4 as compared to pH 4.6. The high charge density led to a decrease in attractive forces while enhancing the repulsive forces between COOH groups, and as a result, high swelling and drug release were perceived at pH 4.6 and 7.4. On other hand, due to protonation at pH 1.2, functional groups of monomers formed conjugates with counterions and thus strengthened the polymeric structure of hydrogel with strong hydrogen bonding. Due to strong hydrogen bonding, low swelling was observed, and thus low drug release was observed [50,51]. Similarly, drug release studies were performed at three pH values of 1.2, 4.6, and 7.4 for the commercially available tablets Theolin S.R (250 mg, PeiLi Pharmaceutical IND. Co., Ltd., Taichung, Taiwan), as shown in Figure 8B. More than 90% drug release was observed for Theolin at pH 7.4 (initial 6 h), 4.6 (initial 8 h), and 1.2 (initial 11 h). 

Like swelling, drug release was also influenced by the various combinations of polymer, monomers, and crosslinker. Drug release was increased with the incorporated compositions of β-CD, Aa, and Ia, as shown in Figure 8C–E. High composition of β-CD, Aa, and Ia led to an increase in generation of –OH, CH_2_OH, and –COOH groups, due to which charge density was increased, and thus an increase in drug release was detected with the increasing composition of polymer and monomers [52,53]. A drop was detected in drug release with the increase in EGDMA composition (Figure 8F). The reason was the high crosslinking and hard network of hydrogel, which retard the penetration of water due to small pore size, and thus a decrease in drug release was observed [54].

Conclusively, we can demonstrate that at the low pH of 1.2, hydrogel remained protonated/un-ionized due to the pKa values of its reagents, and thus low swelling and drug release were observed. As the pH changed from 1.2 to 4.6 and 7.4, deprotonation/ionization of the functional groups of the polymer and monomers started and hydrogel networks expanded, and thus an increase in swelling and drug release was observed. 

The literature indicates that different drug carrier systems have been developed for the sustained/controlled delivery of theophylline. Khan and his coworkers prepared gastroretentive floating tablets of theophylline using hydrophilic polymer METHOCEL K4M and reported release of theophylline for 8 h [55]. Bashir et al. (2016) prepared N-succinyl chitosan-g-poly(methacrylic acid) hydrogels and demonstrated swelling and in vitro release of theophylline for 10–11 h [56]. Similarly, Liu and his coworkers developed pH-sensitive hydroxypropyl methylcellulose acetate succinate based composite nanofibers for controlled delivery of theophylline for up to 12 h [57]. In the current study, the authors reported swelling and in vitro release of theophylline for 72 and 36 h, respectively. Comparing the drug released data of the commercial product Theolin and previously published research work with the present newly prepared hydrogels, we can conclude that release of theophylline was sustained for a long time by fabricated hydrogels in a controlled way. Thus, the newly prepared hydrogels can be considered as one of the most suitable and promising carriers for controlled drug delivery.

### 2.12. Kinetic Modeling 

The best-fit release kinetic model and release rate of the drug from the fabricated hydrogel were determined from in vitro drug release data of all formulations by considering zero order, first order, Higuchi, and Korsmeyer–Peppas as kinetic models. The best-fit model and drug release mechanism were confirmed from the “r^2^” value and “n” value individually. The “r^2^” values indicate the regression coefficient. Table 3 indicates that all formulations of fabricated hydrogel followed Korsmeyer–Peppas model of kinetics because “r^2^” values of Korsmeyer–Peppas model of all formulations were closer to 1 as compared to other kinetic models. Similarly, the “n” value determines the type of diffusion. If “n” > 0.45, then the diffusion is non-Fickian, whereas if “n” ≤ 0.45, then the diffusion is Fickian. The fabricated hydrogel exhibited non-Fickian diffusion because the “n” values of all formulations were within the range of 0.5316–0.7120 [58,59].

## 3. Conclusions

β-CD-*g*-P(Aa/Ia) hydrogels were prepared successfully by the free radical polymerization technique. The structural configuration of the prepared hydrogel and its contents was evaluated by FTIR analysis. The thermal stability of β-CD and Ia was found to be less than that of formulated hydrogel, as indicated by TGA thermogram. A decrease in crystalline and highly sharp peaks of β-CD and Ia was observed after crosslinking, as revealed by XRD analysis. Similarly, a hard surface was observed by the SEM. Swelling and drug release were found to be higher at pH 7.4 compared to pH 4.6 and 1.2 with an order of pH 7.4 > 4.6 > 1.2, presenting the pH-responsive nature of the fabricated hydrogel. Increased incorporation of β-CD, Aa, and Ia led to an increase in porosity, swelling, drug loading, and drug release, while a decrease was observed with the increased incorporation of EGDMA. A drop in sol fraction was seen with the increasing compositions of hydrogel contents, whereas gel fraction was increased. Polymer volume fractions were higher at low pH 1.2 and lower at high pH 7.4 and 4.6, indicating the maximum swelling capability of the developed hydrogel at high pH values. Thus, we can conclude from the discussion that prepared grafted hydrogel could be applied for controlled delivery of theophylline.

## 4. Materials and Methods

### 4.1. Materials

Theophylline and β-cyclodextrin were purchased from Sigma-Aldrich (St. Louis, MI, USA) and Alfa Aesar (Thermo Fisher Scientific, Ward Hill, MA, USA). Acrylic acid and itaconic acid were obtained from Acros (Carlsbad, CA, USA) and Acros Organics (Janssen Pharmaceuticalaan, Belgium). Similarly, ammonium persulfate was acquired from Showa (Tokyo, Japan), whereas ethylene glycol dimethacrylate was obtained from Alfa Aesar (Tewksbury, MA, USA).

### 4.2. Synthesis of β-CD-g-P(Aa/Ia) Hydrogels

Different formulations of β-cyclodextrin-*graft*-poly(acrylic acid/itaconic acid) (β-CD-*g*-P(Aa/Ia)) hydrogels were formulated by the free radical polymerization technique. An accurate amount of β-CD was dissolved in deionized distilled water. Similarly, ammonium persulfate (APS) was dissolved in deionized distilled water, while Aa was already available in liquid form. A mixture of water and ethanol was used for dissolving Ia at a temperature of 50 °C. APS solution was added into IA solution, and after proper mixing, the solution was poured into β-CD solution. The mixture was stirred for 20 min, and after that, Aa was added dropwise into the stirred mixture. Finally, ethylene glycol dimethacrylate (EGDMA) was added to the polymer and monomer mixture. After 5 min, a transparent solution was formed, which was purged by nitrogen gas in order to remove dissolved oxygen. The transparent solution was transferred into glass molds, which were kept in a water bath at 55 °C for the initial 2 h. The temperature was further increased up to 65 °C for the next 22 h. The prepared gel was sliced into 8 mm size discs. A mixture of water and ethanol was used for washing the gel discs in order to remove any impurity if attached to the surface of the gel discs. After that, the discs were placed at 40 °C in a vacuum oven for complete dehydration after exposing the gel discs to room temperature for 24 h. The dried discs of hydrogel were processed for further investigation. 

### 4.3. Fourier Transform Infrared Spectroscopy (FTIR)

FTIR spectra of β-CD, Aa, Ia, THP, and the unloaded and loaded β-CD-g-P(Aa/Ia) hydrogel were obtained by using attenuated total reflectance FTIR (Nicolet 380 FTIR (Thermo Fisher Scientific, Ishioka, Japan)). Samples were ground thoroughly, and then the FTIR spectrum was analyzed within the range of 4000–500 cm^−1^ [60].

### 4.4. Thermogravimetric Analysis (TGA)

TGA (PerkinElmer Simultaneous Thermal Analyzer STA 8000 (PerkinElmer Ltd.,

Buckinghamshire, UK) was conducted in order to evaluate the thermal stability of β-CD, Ia, and fabricated β-CD-g-P(Aa/Ia) hydrogel. Samples of 0.3 to 5 mg were finely ground and placed in a platinum pan, which was attached with a microbalance. TGA thermogram was performed within the temperature range of 40–600 °C. Heat rate and nitrogen flow were maintained 20 °C/min and 20 mL/min, respectively, throughout the TGA analysis [61]. 

### 4.5. X-ray Diffraction Studies (XRD)

The crystalline or amorphous nature of β-CD, Ia, and fabricated β-CD-g-P(Aa/Ia) hydrogel was evaluated by X-ray diffraction (XRD-6000 Shimadzu, Tokyo, Japan) analysis. The crystallinity of a compound is identified by its sharp peaks, while diffuse peaks indicate the amorphous nature of the compound. The weighed crushed samples were placed in a sample holder and leveled with the help of a glass slide. The XRD analysis was performed within the range of 10–60° with an angle of 2θ 2°/min [62].

### 4.6. Scanning Electron Microscopy (SEM)

SEM (JSM-5300 model, (JEOL, Tokyo, Japan)) was carried out to determine the surface and structural characteristics of the developed hydrogels. Hence, the dried hydrogel disc was mounted on an aluminum point by sticky tape, covering the entire area. With the help of a gold splutter coater, a thin layer of gold was coated in an inert environment while using a vacuum evaporator. Photomicrographs were taken, and thus surface morphology of fabricated hydrogel was evaluated [63].

### 4.7. Sol–Gel Fractions

Sol–gel fractions of fabricated hydrogels were measured with the purpose of evaluating the amount of reactants consumed in their preparation by estimating the sol and gel content. Therefore, a dried hydrogel disc of known weight was placed in Soxhlet apparatus containing deionized distilled water. The extraction process was carried out at a temperature of 85 °C for 10 h. After that, the disc was removed and placed in a vacuum oven at 40 °C until dryness. The dried disc was weighed again [64]. Sol and gel fractions were estimated by using the following formulas:

(1) $$\mathrm{Sol}\;\mathrm{fraction}\;\%=\frac{J_{1}-J_{2}}{J_{2}}\times 100$$

(2) Gel fraction=100−Sol fraction

where J_1_ is the initial weight of dried hydrogel disc before the extraction process and J_2_ is the final weight after the extraction. 

### 4.8. Porosity Study

A porosity study was conducted for all formulations of fabricated β-CD-g-P(Aa/Ia) hydrogels. Porosity was determined by the solvent displacement method. Absolute ethanol was employed as a displacement solvent. Thus, weighed dried hydrogel discs (M_1_) were immersed for 72 h in absolute ethanol. After that, discs were taken out, blotted with filter paper to remove excess ethanol attached to the surface of the discs, and weighed again (M_2_) [65]. The following formula was employed for the determination of (%) porosity:

(3) $$(\%)\;\mathrm{Porosity}=\frac{M_{2}-M_{1}}{\rho V}\times 100$$

where ρ indicates the density of absolute ethanol and V represents the swelling volume of hydrogel discs. 

### 4.9. Swelling Study

A swelling study was performed for the developed hydrogels at pH 1.2, 4.6, and 7.4, at 37 °C in order to determine their pH-responsive nature. Therefore, a dried hydrogel disc of known weight was immersed in the respective 100 mL buffer solutions. After a regular interval of time, the disc was removed, blotted with filter paper, weighed again, and immersed back in the respective buffer medium. This process was continued until an equilibrium weight was achieved [66]. This study was performed in a triplicate. Dynamic swelling was estimated by using the following formula:

(4) $$\left(q\right)=\frac{N_{2}}{N_{1}}$$

where q shows the dynamic swelling, N_1_ represents the initial weight of the dried hydrogel disc before swelling, and N_2_ indicates the final weight after swelling at time t.

### 4.10. Polymer Volume Fraction

Polymer volume fraction indicates the fraction of polymer in a completely swelled state and is represented by V2,s. Equilibrium volume swelling (Veq) data of the formulated hydrogels at three different pH values (1.2, 4.6, and 7.4) were employed for the estimation of polymer volume fraction [47]. Therefore, the following formula was used for the determination of polymer volume fraction:

(5) $$V2,s=\frac{1}{V_{eq}}$$

### 4.11. Drug Loading 

The diffusion and absorption method was used for drug loading of the developed hydrogels. Hence, a 1% drug (THP) solution was formed in a phosphate buffer solution of pH 7.4. Weighed dried hydrogel discs were immersed in the drug solution for 72 h. After achieving equilibrium swelling, discs were removed, washed with distilled water to remove any excess entrapped drug on the surface of hydrogel discs, and then placed in a vacuum oven at 40 °C for dryness. The dried hydrogel discs were weighed again. This experiment was performed in a triplicate.

Quantification of the drug loaded by the developed hydrogel was estimated by weight and extraction methods. In weight method, the weight of the unloaded dried hydrogel disc was subtracted from the drug-loaded dried hydrogel disc as indicated in the following formula:

Drug loaded quantity = X_L_ − X_UL_ (6)

where X_L_ represents the weight of the drug-loaded hydrogel discs and X_UL_ indicates the weight of the unloaded hydrogel discs. 

In the extraction method, a weighed dried disc of hydrogel was taken and immersed in a 25 mL phosphate buffer solution of pH 7.4. After a regular interval of time, samples were collected, and the buffer was replaced each time by a fresh medium of the same concentration. The process was continued until the entire drug was eliminated completely from the hydrogel disc. The collected samples were then evaluated on a UV-Vis spectrophotometer (U-5100,3J2-0014, Tokyo, Japan) at λ_max_ 272 nm, and hence drug content was determined [67]. 

### 4.12. Drug Release Studies

In vitro drug release studies were carried out to evaluate the drug release from commercially available tablets, Theolin S.R (250 mg, PeiLi Pharmaceutical IND. Co., Ltd.), and developed hydrogels at three various pH values, i.e., pH 1.2., 4.6 and 7.4. Hence, Theolin and loaded dried discs of hydrogel were immersed individually in 900 mL phosphate buffer solutions of pH 1.2, 4.6, and 7.4 in a USP dissolution apparatus type II (USP dissolution (Sr8plus Dissolution Test Station, Hanson Research, Chatsworth, CA, USA)) at 37 ± 0.5 °C and 50 rpm. An aliquot of 5 mL was taken periodically and replenished with fresh medium of the same concentration in order to keep the sink condition constant. The collected samples were then filtered and analyzed on a UV-Vis spectrophotometer (U-5100,3J2-0014, Tokyo, Japan) at λ_max_ 272 nm in a triplicate [36].

### 4.13. Kinetic Modeling 

Various kinetic models such as zero order, first order, Higuchi, and Korsmeyer–Peppas were evaluated by fitting the achieved in vitro drug release data in order to determine the order and release mechanism of the drug from the fabricated hydrogels [68].

### 4.14. Statistical Analysis

SPSS Statistics software 22.0 (IBM Corp, Armonk, NY, USA) was employed for the statistical analysis. Student’s *t*-test was used for the determination of variations between the tests, which were found statistically significant when the achieved p-value was less than 0.05.

## Figures and Tables

**Table 1:** Feed ratio scheme for the formulation of β-CD-*g*-P(Aa/Ia) hydrogels.

F. Code	Polymer β-CD g/100 g	Monomer Aa g/100 g	Monomer Ia g/100 g	Initiator APS g/100 g	Crosslinker EGDMA g/100 g
BAIF-1	0.5	20	10	0.5	1.5
BAIF-2	1.0	20	10	0.5	1.5
BAIF-3	1.5	20	10	0.5	1.5
BAIF-4	0.5	20	10	0.5	1.5
BAIF-5	0.5	25	10	0.5	1.5
BAIF-6	0.5	30	10	0.5	1.5
BAIF-7	0.5	20	05	0.5	1.5
BAIF-8	0.5	20	10	0.5	1.5
BAIF-9	0.5	20	15	0.5	1.5
BAIF-10	0.5	20	10	0.5	1.0
BAIF-11	0.5	20	10	0.5	1.5
BAIF-12	0.5	20	10	0.5	2.0

**Table 2:** Polymer volume fraction and drug loading of β-CD-*g*-P(Aa/Ia) hydrogels.

Formulation Code	Polymer Volume Fraction			Drug Loaded (mg)/500 mg of Dry Gel	
	pH 1.2	pH 4.6	pH 7.4	Weight Method	Extraction Method
BAIF-1	0.454	0.260	0.156	81.8 ± 0.8	80 ± 1
BAIF-2	0.363	0.250	0.150	104.4 ± 1	103.2 ± 1
BAIF-3	0.248	0.242	0.147	116.3 ± 1	115.6 ± 1
BAIF-4	0.454	0.260	0.156	81.8 ± 0.8	80 ± 1
BAIF-5	0.352	0.245	0.146	110.1 ± 1	109.5 ± 1
BAIF-6	0.240	0.236	0.138	121.2 ± 1	119.7 ± 1
BAIF-7	0.470	0.270	0.163	75.4 ± 1	73.6 ± 1
BAIF-8	0.454	0.260	0.156	81.8 ± 0.8	80 ± 1
BAIF-9	0.434	0.255	0.151	86.7 ± 0.9	85.3 ± 1
BAIF-10	0.416	0.245	0.128	88.2 ± 1	88 ± 1
BAIF-11	0.454	0.260	0.156	81.8 ± 0.8	80 ± 1
BAIF-12	0.532	0.280	0.184	70.3 ± 1	69.1 ± 1

**Table 3:** Kinetic modeling release of THP from β-CD-*g*-P(Aa/Ia) hydrogels.

F. Code	Zero Order r^2^	First Order r^2^	Higuchi r^2^	Korsmeyer–Peppas r^2^ n	
BAIF-1	0.9661	0.9283	0.9355	0.9843	0.5316
BAIF-2	0.9825	0.9894	0.9695	0.9963	0.6073
BAIF-3	0.9846	0.9952	0.9879	0.9986	0.6930
BAIF-4	0.9661	0.9283	0.9355	0.9843	0.5316
BAIF-5	0.9740	0.9795	0.9793	0.9893	0.6437
BAIF-6	0.9881	0.9649	0.9670	0.9980	0.7120
BAIF-7	0.9787	0.9081	0.9142	0.9822	0.6560
BAIF-8	0.9661	0.9283	0.9355	0.9843	0.5316
BAIF-9	0.9768	0.9763	0.9806	0.9810	0.6245
BAIF-10	0.9803	0.8426	0.9218	0.9874	0.6173
BAIF-11	0.9661	0.9283	0.9355	0.9843	0.5316
BAIF-12	0.9545	0.9141	0.9681	0.9794	0.6989
